# Characterization of Clinical and Environmental *Mycobacterium avium* Spp. Isolates and Their Interaction with Human Macrophages

**DOI:** 10.1371/journal.pone.0045411

**Published:** 2012-09-19

**Authors:** Evelyn Guirado, Jesus Arcos, Rose Knaup, Rebecca Reeder, Bret Betz, Cassie Cotton, Tejal Patel, Stacy Pfaller, Jordi B. Torrelles, Larry S. Schlesinger

**Affiliations:** 1 Center for Microbial Interface Biology, The Ohio State University, Columbus, Ohio, United States of America; 2 Departments of Microbial Infection and Immunity, and Internal Medicine, The Ohio State University, Columbus, Ohio, United States of America; 3 United States Environmental Protection Agency, Cincinnati, Ohio, United States of America; Institut de Pharmacologie et de Biologie Structurale, France

## Abstract

Members of the *Mycobacterium avium* complex (MAC) are naturally occurring bacteria in the environment. A link has been suggested between *M. avium* strains in drinking water and clinical isolates from infected individuals. There is a need to develop new screening methodologies that can identify specific virulence properties of *M. avium* isolates found in water that predict a level of risk to exposed individuals. In this work we have characterized 15 clinical and environmental *M. avium* spp. isolates provided by the US Environmental Protection Agency (EPA) to improve our understanding of the key processes involved in the binding, uptake and survival of these isolates in primary human macrophages. *M. avium* serovar 8 was predominant among the isolates studied. Different amounts and exposure of mannose-capped lipoarabinomannan (ManLAM) and glycopeptidolipids (GPLs), both major mycobacterial virulence factors, were found among the isolates studied. Reference clinical isolate 104 serovar 1 and clinical isolates 11 and 14 serovar 8 showed an increased association with macrophages. Serum opsonization increased the cell association and survival at 2 h post infection for all isolates. However, only the clinical isolates 104 and 3 among those tested showed an increased growth in primary human macrophages. The other isolates varied in their survival in these cells. Thus we conclude that the amounts of cell envelope ManLAM and GPL, as well as GPL serovar specificity are not the only important bacterial factors for dictating the early interactions of *M. avium* with human macrophages.

## Introduction

The *M. avium* complex (MAC) comprises a heterogeneous group of slow-growing mycobacteria. Two distinct species within MAC are *M. avium* and *M. intracellulare.* Contrary to *M. intracellulare*, which relates to a single species, *M. avium* consists of at least five subspecies according to 16S RNA analysis, including *M. avium* subsp. *avium*, subsp. *hominissuis,* subsp. *silvaticum*, subsp. *lepraemurium* and subsp. *paratuberculosis* (with its ovine, bovine, and caprine types) [1].

MAC organisms are ubiquitous. In general, these organisms are found in several natural ecosystems, such as soil, dust, vegetation, and salt and fresh water [2]–[4]. MAC bacteria are considered opportunistic pathogens in AIDS patients as well as a source of pulmonary infections in non-AIDS patients. Although disease in people with normal immunity is usually self-limited, pulmonary MAC infections appear to have increased during recent decades [5], [6]. In the past, genetic relationships between MAC species found in the environment and those found in human infections have been difficult to establish [7], [8]. However recently a link has been suggested between MAC isolates in drinking water and clinical isolates from infected individuals [3], [9]; although the proportion of disease associated with exposure remains unknown. In this context, MAC can be harbored in shower heads [10], where the amounts found can be 100-fold higher than those found in the associated water supply. Since MAC has been shown to be chlorine resistant [11], municipal water supply treatments may actually be enriching its relative abundance.

Not only susceptibility of the host but also virulence properties of these bacteria contribute to their pathogenicity. In this context, many cell wall glycolipids are considered to be virulence factors in mycobacterial pathogenesis. Among them, glycopeptidolipids (GPLs) play a major role in MAC [12], [13] and their absence or modification corresponds with an attenuated phenotype [14].

GPLs are classified as the serovar (Ser.)-specific, polar GPLs (ssGPLs) and non-specific, apolar GPLs (nsGPLs) [15]. The basic structure of the GPLs is given by the nsGPLs, which have an invariable tripeptide amino alcohol core modified with an amide-linked β-hydroxy fatty acid, an *O*-linked di-methylated rhamnose and a 6-deoxy-talose. In the case of the ssGPLs, the 6-deoxy-talose residue can be further linked to an array of sugars that vary in length and composition which define the specific MAC serovar [16].

GPLs have been shown to play a role in host-pathogen interactions, affecting the initial or long-term response of the host. MAC bacteria undergo receptor-mediated phagocytosis by macrophages and survive in a mycobacterial phagosome [1]. In this context, some studies suggest that GPLs can interact with host membranes promoting bacterial survival [17]. In addition, GPLs can accumulate on the surface of MAC during growth [18] and inside infected cells [19], [20]. These data together with evidence for an extracellular ‘capsule-like’ material on MAC predominantly formed by GPLs indicate that these glycolipids serve as a protective barrier against the host cell-mediated immune response in the phagosome [20]. GPLs can also delay phagolysosomal fusion, an effect that is, in part, dependent on the macrophage mannose receptor (MR) [21]. Another cell wall component that has been implicated in delaying phagolysosomal fusion is the surface-exposed mannose-capped lipoarabinomannan (ManLAM). As in the case of the GPLs, ManLAM-mediated limitation of phagolysosomal fusion is dependent on the MR [22]. ManLAM in MAC is mono-capped with a single mannose on its non-reducing termini; whereas ManLAM in *Mycobacterium tuberculosis* (*M.tb*) contains mainly di- and tri-caps with 2 to 3 mannoses [23].

In this study we characterized several clinical and environmental *M. avium* spp. isolates provided by the US Environmental Protection Agency (EPA) using well established analytical biochemistry tools and studied the interaction of bacteria with human macrophages in an attempt to correlate GPL and ManLAM cell wall content with the capacity to infect and survive within human macrophages *in vitro*.

## Materials and Methods

### Chemicals, Reagents and Bacterial Isolates

All chemicals used in this study were of the highest purity available from Sigma-Aldrich unless otherwise specified. The monoclonal antibody (mAb) against LAM (CS-35) was kindly provided by both the Tuberculosis Research Materials and Vaccine Testing contract (NOI-AI-75320) and Leprosy Research Support contract (NOI-AI-25469) located at Colorado State University. The 15 clinical and environmental *M. avium* spp. isolates genotyped by nucleic acid hybridization test (AccuProbe) and by amplified fragment length polymorphism [24] were provided by the US Environmental Protection Agency (EPA). The clinical isolate MAC 104, used as the reference isolate [25], was kindly provided by Dr. Andrea Cooper (Trudeau Institute, Saranac Lake, NY). For each experiment, aliquots of MAC frozen stocks were plated on 7H11 agar plates (Difco, Detroit, MI) enriched with 5% oleic acid-albumin-dextrose-catalase (OADC) and bacteria were grown for 9–21 days at 37°C in 5%CO_2_.

### Extraction of GPLs

GPLs were obtained as we previously described [26]. Briefly, bacilli were scraped from agar plates and transferred directly to sterile screw-capped glass culture tubes and suspended in chloroform:methanol (2∶1, v/v) at a cell:volume ratio of approximately 1∶10. Cell wall lipids were extracted at 37°C for 12 h, and the lipid-containing organic layer was removed by centrifugation at 2,100×*g* for 10 min. This step was repeated once, and organic layers were combined. Extracted total lipids were then treated with mild-alkaline hydrolysis (0.2 M NaOH in methanol at 37°C for 40 min) to purify the alkaline resistant GPLs. After pH neutralization with acetic acid, the organic layer was dried down, weighed, and suspended in chloroform:methanol (2∶1, v/v) at a known concentration. Purified GPLs were applied to thin layer chromatography (TLC) plates, developed in chloroform:methanol:water (30∶8:1, v/v/v), sprayed with a solution of α-naphthol in 5% sulfuric acid in ethanol, and visualized by heating the plates at 100°C to detect the characteristic pink-purple color as a result of the inherent 6-deoxy-hexoses. Total lipid extracts were also directly spotted onto TLC plates, developed as above and detected by spraying with a solution of 10% sulfuric acid in water followed by heating at 100°C.

### Neutral Sugar Analysis

Total GPL extractions (100 µg) were converted to alditol acetates using *scyllo*-inositol as an internal standard and analyzed by gas chromatography/mass spectrometry (GC/MS) as we previously described [26]. GC of alditol acetates was performed on a ThermoQuest Trace Gas Chromatograph 2000 connected to a GCQ/Polaris MS detector (ThermoQuest, Austin, TX) at an initial temperature of 50°C for 1 min, increasing to 170°C at 30°C/min and finally to 270°C at 5°C/min. All experiments were performed in duplicate using three independent GPL extracts.

### Extraction of Lipomannan/ManLAM

ManLAM and lipomannan (LM) extractions were performed as previously described [27]. Briefly, bacteria were scraped from agar plates and delipidated using chloroform:methanol (2∶1, v/v) followed by chloroform:methanol:water (10∶10:3, v/v/v) for 48 h at 37°C with constant shaking. The residual delipidated biomass was dried, suspended in endotoxin-free PBS and lysed using a FP-120 bead beater applying a total of seven cycles (1 min-on/1 min-off). Lysate protein quantification was performed by the BCA method before treatment with proteinase K (2 mg/ml) at 37°C for 18 h. After proteinase K treatment, the reaction was centrifuged at 27,000 × g for 10 min, and the resulting supernatant dialyzed against MQ-H_2_O using a 3,500 Da molecular mass cut off membrane for 24 h. The retentate was then dried and analyzed by 15% SDS–PAGE followed by periodic acid-silver (PAS) staining of the gel to visualize the extracted ManLAM and LM [27]. Immunoblotting using anti-LAM CS-35 mAb was performed essentially as previously described [28]. Densitometry analyses on the blots were performed using image Acquisition and Analysis Labworks software version 4.6 (UVP Bioimaging system, UVP Inc, Upland, CA).

### Analysis of Surface Exposed ManLAM on *M.avium* Spp. Isolates by Whole Cell ELISA

Assessment of ManLAM surface exposure on whole bacteria was performed as we previously described [29]. Briefly, 2×10^6^ bacteria/well were dried down using a 96-well format, and blocked with PBS containing 1% bovine serum albumin (BSA) in 0.05% aqueous Tween 20 (blocking buffer) for 2 h at room temperature (RT). Blocked wells were washed several times with PBS containing 0.05% aqueous Tween 20 (washing buffer) and incubated with CS-35 mAb in blocking buffer overnight at RT. The wells were then washed several times with washing buffer and incubated with HRP-conjugated goat anti-mouse IgG (Bio-Rad) in blocking buffer for 2 h at RT. Finally, wells were washed several times using PBS and developed using an HRP-substrate kit (Bio-Rad) and stopped following the manufacturer’s instructions. Antibody reactivity was measured at an absorbance of 405 nm. An isotype control Ab was used in preliminary experiments and showed no background.

**Figure 1:**
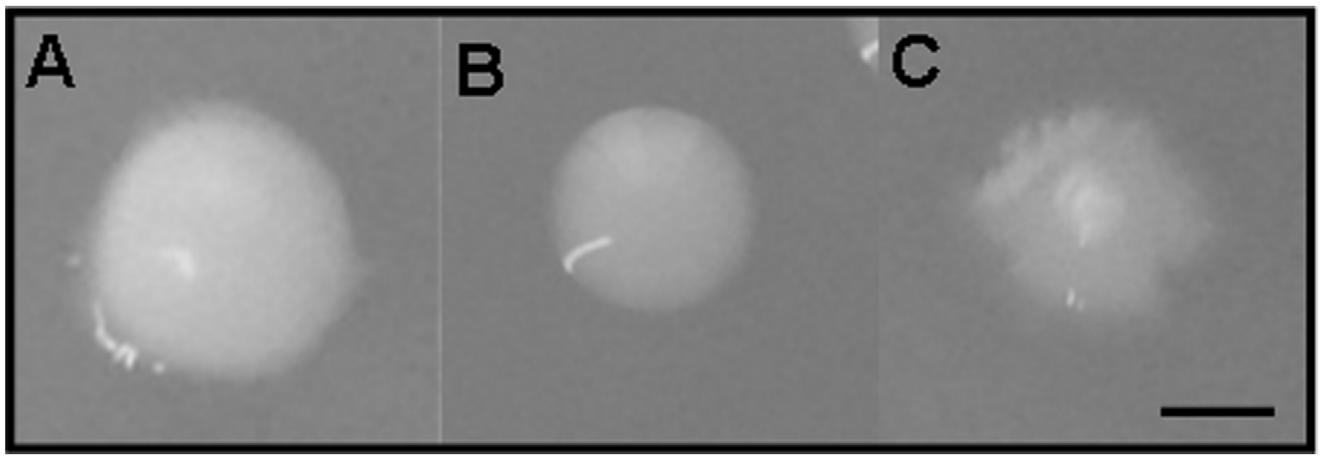
Colony morphotypes of *M. avium* spp. isolates. Photographs show representative colony morphotypes of MAC isolates grown on 7H11+OADC plates after 21 days of incubation at 37°C in 5% CO_2_. **A**. Smooth, opaque, regular and white morphotype (phenotype observed for all isolates except 2 and 13). **B**. Smooth, transparent and regular morphotype (phenotype observed for isolates 5, 10, 11, 12, 13 and 104). **C.** Smooth, transparent and irregular (phenotype observed for isolates 5, 10, 11, 12 and 104). Bar indicates 2 mm. The photographs are representative of N = 3.

**Table 1:** Source and colony morphotype classification of MAC isolates.

Isolate	Source	Colony morphotype
1	AIDS patient	Smooth, opaque, regular, white
2	Domestic tap water	Dry, opaque, irregular, white
3	AIDS patient	Smooth, opaque, regular/irregular, white/yellow
4	AIDS patient	Smooth, opaque, regular, white
5	AIDS patient	Smooth, opaque/transparent, regular/irregular, white/yellow
6	AIDS patient	Smooth opaque, regular, white
7	Hospital 13	Smooth, opaque, regular/irregular, white
8	Italian brown mushrooms	Smooth, opaque, regular, white
9	Domestic tap water	Smooth, opaque, regular, white
10	Domestic tap water	Smooth, opaque/transparent, regular/irregular, white
11	Hospital 7	Smooth, opaque/transparent, regular/irregular, white/yellow
12	Spinach	Smooth, opaque/transparent, regular/irregular, white
13	Sprouts	Smooth, transparent, regular, white
14	Non-AIDS patient	Smooth, opaque, regular, white
15	Domestic tap water	Smooth, opaque, regular, white
104	AIDS patient	Smooth, opaque/transparent, regular/irregular, white/yellow

### Analysis of Surface Exposed ManLAM on *M. avium* Spp. Isolates by Flow Cytometry

Assessment of ManLAM surface exposure was performed as we previously described [29]. Live single cell suspensions (1×10^6^) were blocked with 2% BSA in PBS, washed with PBS followed by staining with anti-LAM CS-35 in 2% BSA in PBS for 20 min at 4°C. Samples were washed with PBS and stained with Alexa Fluor 488 goat anti-mouse IgG Ab (life technologies). After further washing, samples were processed by FACSDiva and results were analyzed by FlowJo software. Mean fluorescence intensity (MFI) was measured for each isolate and the average values of 2 samples were obtained. An isotype control Ab was used in preliminary experiments and showed no background.

### Isolation and Preparation of Human Macrophage Monolayers

Human monocyte-derived macrophage (MDM) monolayers were prepared from healthy tuberculin-negative human volunteers (using an approved Institutional Review Board protocol at The Ohio State University) as we previously described [30]. Written informed consent was provided by study participants and/or their legal guardians. Monolayers (2×10^5^ MDMs) for association and survival assays were obtained by adherence to acid-washed glass coverslips or directly to plastic, respectively, in 24-well tissue culture plates for 2 h at 37°C in 5% CO_2_.

### Preparation of *M. avium* Spp. Isolates for Macrophage Studies

Single cell suspensions for each isolate studied were prepared as we previously described [31]. Briefly, bacteria were scraped from agar plates, suspended in RPMI-20 mM HEPES, briefly vortexed (five pulses) with two glass beads (3 mm), and allowed to settle for 30 min. The upper bacterial suspension (devoid of clumps) was then transferred to a second tube and let rest for an additional 5 min to obtain the final single cell suspension. Bacterial concentrations were determined by counting using a Petroff-Hausser chamber and subsequent plating for CFUs.

**Figure 2:**
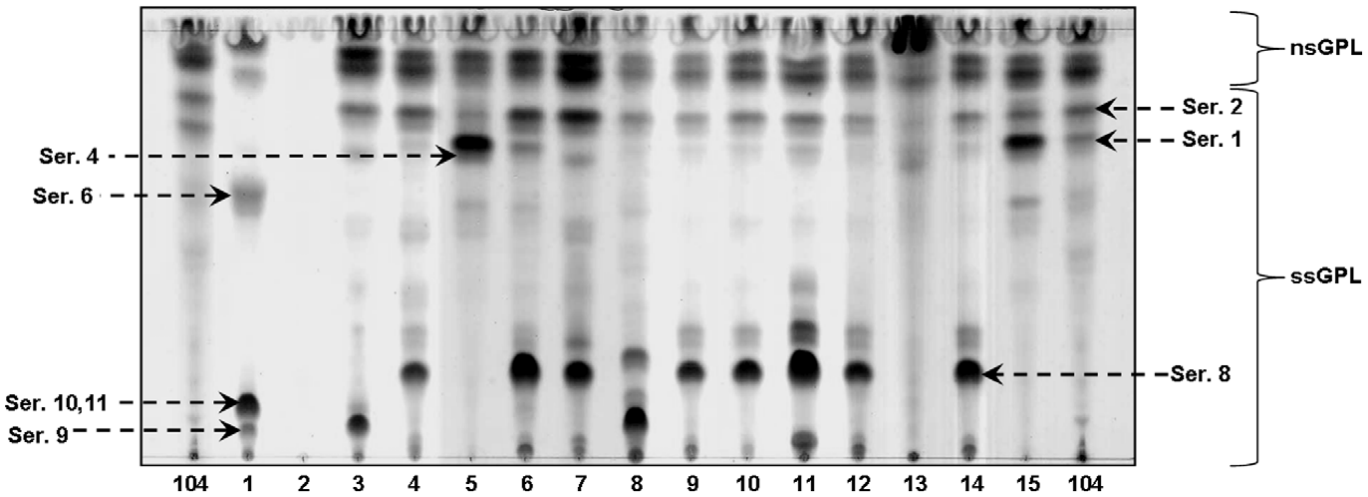
TLC of *M. avium* spp. isolates. GPLs from each *M. avium* spp. isolate were purified from total lipid extracts. Total GPL profiles were examined by TLC using chloroform:methanol:water (30∶8:1, v/v/v) as the solvent system and α-naphthol as the developer. Serovars were assigned based on a previous publication [25]. Serovar-specific polar GPLs (ssGPLs); serovar non-specific apolar GPLs (nsGPLs). Representative experiment of N = 3.

**Table 2:** Percentage of GPLs extracted.

Isolate	Total lipidweight (mg)	GPLs weight(mg)	%GPLs
1	24.55	16.08	65
2	7.35	4.32	59
3	28.06	15.28	54
4	20.83	13.27	64
5	22.50	14.43	64
6	20.56	12.54	61
7	18.52	6.42	35
8	17.11	9.79	57
9	16.68	9.58	57
10	16.46	10.23	62
11	2.53	0.78	31
12	12.42	6.38	51
13	10.51	2.84	27
14	16.02	8.87	55
15	25.09	14.91	59
104	211.20	65.57	31

### *M. avium* Spp. Association with and Intracellular Growth in Human Macrophages

Assessment of association with and intracellular growth in human macrophages was performed as we previously described [30], [32]. Briefly, day 12 MDM monolayers in RPMI (BD Biosciences) containing 20 mM HEPES (RH) and 10% autologous human serum or RPMI containing 20 mM HEPES and 1 mg/ml human serum albumin (RHH) were incubated with single cell suspensions of bacilli at a multiplicity of infection (MOI) of 25∶1 for association; or a MOI of 5∶1 for survival, for 2 h at 37°C in 5% CO_2_. In certain experiments, MDM monolayers were also pre-incubated with 2.5 mg/ml of mannan for 20 min at 37°C to evaluate specific association through the macrophage MR as we previously described [33].

For association assays, after the 2 h infection period, MDM-infected monolayers were washed several times with warm RPMI to remove non-associated bacilli, fixed in 10% aqueous formalin, and finally washed again several times with PBS to remove residual fixative. Associated bacilli were stained with auramine-rhodamine and the number of cell-associated bacilli/MDM on triplicate coverslips was determined by counting >300 consecutive MDMs/coverslip using phase-contrast fluorescence microscopy as we previously described [30]. Association assays of the isolates with MDMs were performed in triplicate using a minimum of 3 independent donors.

**Figure 3:**
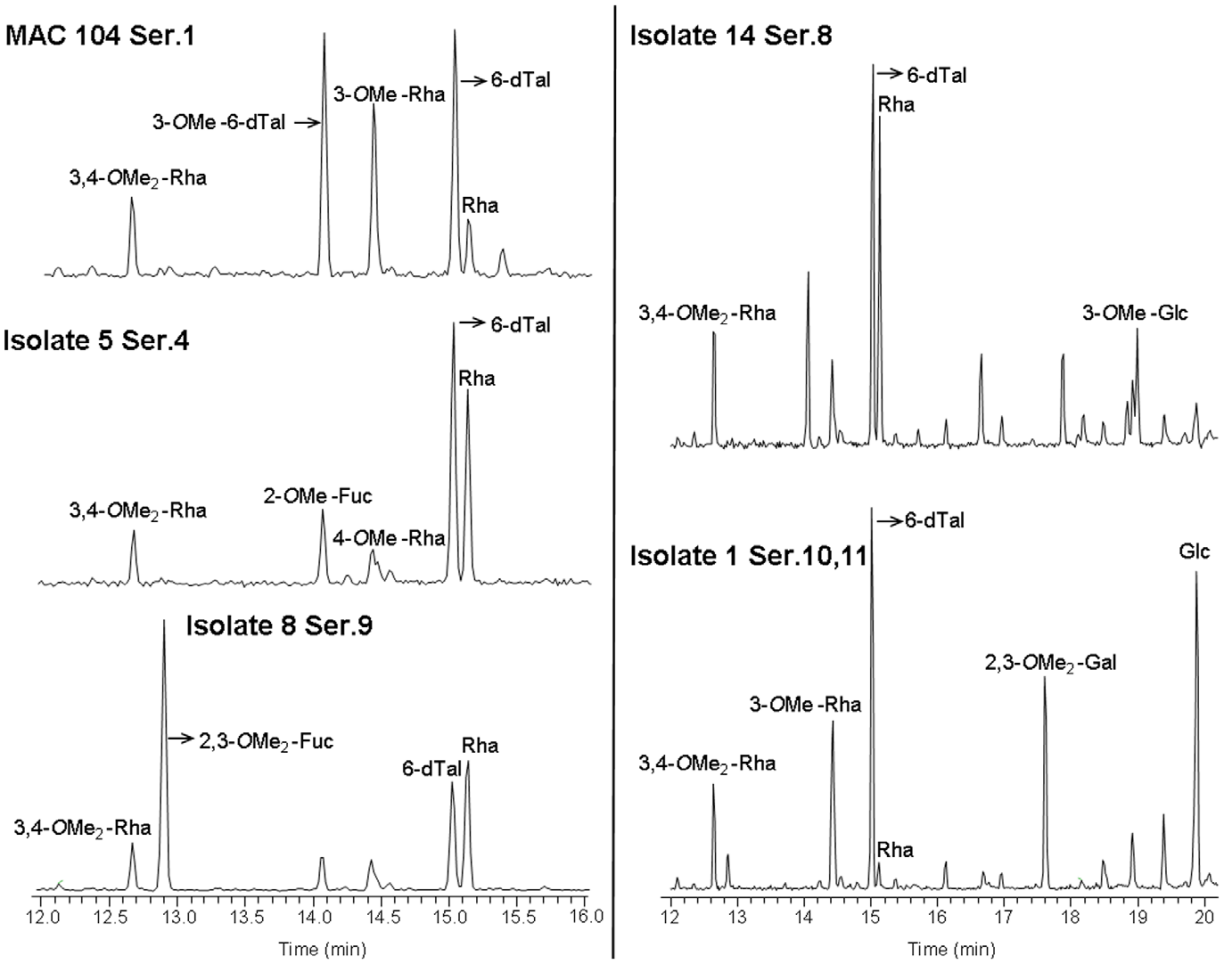
GC profiles of purified GPLs from *M. avium* spp. 1, 5, 8, 14 and 104. GPLs were hydrolyzed using 2 M trifluoroacetic acid, reduced and acetylated. Aditol acetate GC profiles were obtained and peaks were identified by MS analyses. Shown are representative plots for each serovar (N = 3). Note that the horizontal axis for isolates 104, 5 and 8 (left side of figure) differs from isolates 14 and 1. Methyl (Me); Rhamnose (Rha); Talose (Tal); Fucose (Fuc); Galactose (Gal); Glucose (Glc).

To Assess Intracellular Growth, MDM-infected Monolayers were Washed and Either Repleted with RH Containing 1% Human Autologous Serum and Further Incubated for 48 to 96 h or directly lysed (2 h time-point) as we previously described [32]. Briefly, after the first time point, supernatants (containing detached infected MDMs) and monolayers were lysed separately and then combined. Lysates were diluted, plated on agar plates, and CFUs determined after incubation for 21 days at 37°C in 5% CO_2_. Bacterial growth assays in MDMs were performed in triplicate using a minimum of 3 different donors.

### Statistics

Prism software (GraphPad 5.0) was used to determine the statistical significance of differences in the means of experimental groups with unpaired, two-tailed Student t tests. P<0.05 was considered significant.

**Table 3:** Confirmed serovar designation of MAC isolates according to TLC and GC/MS.

Isolate	TLC	GC/MS	Remarks
1	6, 9, 10, 11	10, 11	Conclusively Ser. 10, 11
2	–	–	Not a MAC
3	1, 2, 9	9	Conclusively Ser. 9
4	1, 2, 8	1	Conclusively Ser. 1
5	1, 2, 4, 6	4	Conclusively Ser. 4
6	1, 2, 4, 8	1	Conclusively Ser. 1
7	2, 8	8	Conclusively Ser. 8
8	1, 2, 9	9	Conclusively Ser. 9
9	2, 8	8	Conclusively Ser. 8
10	2, 8	8	Conclusively Ser. 8
11	2, 8	8	Conclusively Ser. 8
12	2, 8	8	Conclusively Ser. 8
13	4	4	Conclusively Ser. 4
14	2, 8	8	Conclusively Ser. 8
15	1, 2, 4, 6	4	Conclusively Ser. 4
104	1, 2	1	Conclusively Ser. 1

## Results

### Colony Morphology of *M. avium* Spp. Isolates

Each isolate was plated on 7H11+ OADC agar plates for 21 days at 37°C to assess colony morphotypes. At day 21, the colony types most frequently seen were moist, smooth, opaque, with a regular circular or slightly irregular undulated margin, from white to yellow colonies, which appeared in isolates 1, 3, 4, 6, 8, 9, 14 and 15 (Fig. 1 and Table 1). While most isolates appeared to remain as domed colonies over time, isolates 3, 5 and 7 tended to become dryer, opaque and white or cream color which turned into a darker or more yellowish color. Besides producing the above mentioned colony phenotypes, isolates 5, 10, 11, 12 and the reference clinical isolate 104 also generated a few smooth, transparent colonies with irregular margins (Table 1). Isolate 13 was particularly noted for producing moist, smooth, transparent colonies with regular circular margins (Table 1). Surprisingly, isolate 2 did not behave like the rest of isolates because its growth was faster, its appearance was drier and the colonies were bigger (Table 1). In general, although all colonies reached a few millimeters in diameter, smooth transparent colonies consistently grew in size at a slower rate.

**Figure 4:**
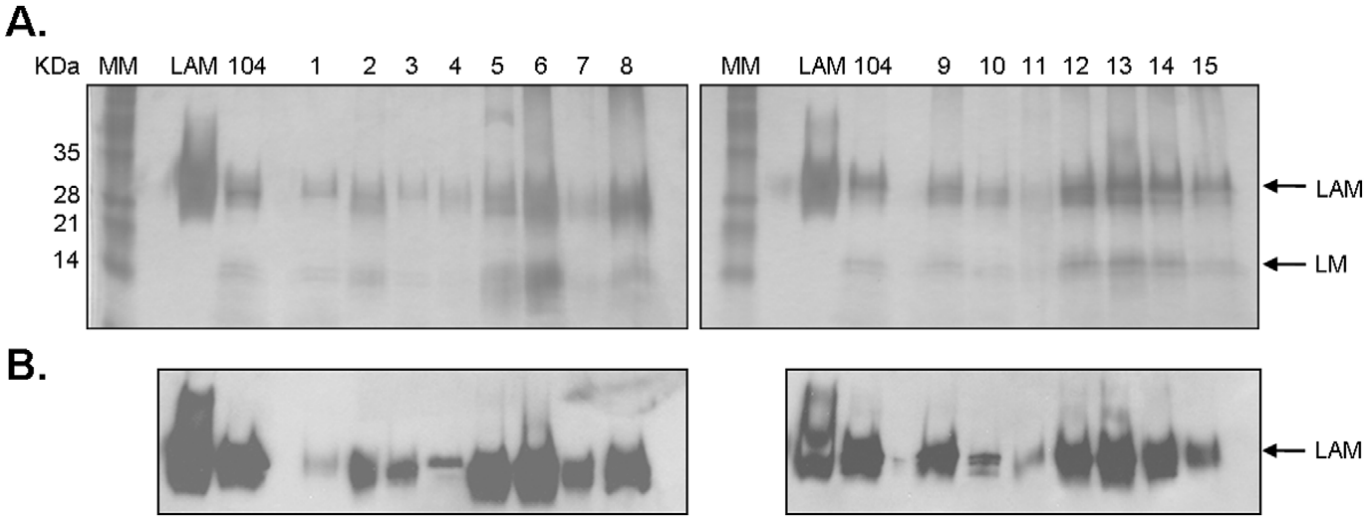
Detection of ManLAM in the cell wall of *M. avium* spp. isolates. A. PAS-stained 15% SDS-polyacrylamide gel of whole cell lysates normalized by protein content (representative of N = 3). *M.tb* H_37_R_v_ ManLAM was used as a control and clinical isolate MAC 104 as the reference standard; lane MM represents a pre-stained protein ladder. **B.** Western blot using anti-LAM CS-35 mAb (representative of N = 3).

### Determination of *M.avium* Spp. Isolate serovars

GPLs were purified from the extracted total lipid fraction obtained from each isolate studied (Table 2). Isolates 1, 4, 5, 6 and 10 showed the highest content of total GPLs based on a percentage of total lipids (>60%) while isolates 7, 11, 13 and reference clinical isolate 104 showed the lowest content of GPLs in their cell wall. We next determined the specific serovar for each isolate by directly analyzing the GPL that defines the serovar by TLC. The specific serovar assignment for each isolate was performed based on their R_f_ values and previous literature [26]. The results showed that all isolates, except 2, contain nsGPLs and ssGPLs (Fig. 2). Based on our previous reports [25], [26] and the limitations that TLC analysis presents [26], a tentative determination of the serovar for each isolate studied was performed. The results indicated that isolate 1 could be a mixture of Ser. 6, Ser. 9 and Ser. 10/11; isolates 3 and 8 could be a mixture of Ser. 1, Ser. 2 and Ser. 9; isolate 4 could be a mixture of Ser. 1, Ser. 2, and Ser. 8; isolates 5 and 15 could be a mixture of Ser. 1, Ser. 2, Ser.4 and Ser. 6; isolate 6 could be a mixture of Ser. 1, Ser. 2, Ser.4 and Ser. 8; isolates 7, 9, 10, 11, 12 and 14 could be a mixture of Ser. 2 and Ser. 8, and isolate 13 was defined as Ser. 4. Reference MAC 104 was identified as Ser. 1 as we previously described [25].

**Table 4:** Detection of ManLAM on the bacterial surface by whole cell ELISA.

Isolate	Mean ± SEM
1	0.189±0.0188
2	0.198±0.0191
3	0.335±0.0534*
4	0.239±0.0331
5	0.2±0.0208
6	0.196±0.0146
7	0.221±0.0349
8	0.189±0.0091
9	0.214±0.0249
10	Below background
11	0.177±0.0127
12	0.245±0.0638
13	0.298±0.0548
14	0.231±0.0265
15	0.319±0.0432*
104	0.181±0.0172
PBS	0.17±0.0125

To confirm the tentative TLC results we directly analyzed the ssGPL sugar composition of the oligosaccharide that defines the serovar by GC/MS, which allowed us to precisely determine the serovar. GPLs were hydrolyzed using 2 M trifluoroacetic acid, reduced and acetylated as described in the materials and methods section. Alditol acetate GC profiles were obtained and peaks were identified by MS analyses. Results (Fig. 3) showed that isolate 1 had the fragment ions of 3-*O*-methyl-rhamnose (Retention time [R_t_]: 14.43: *m/z* 130, 143, 190, 203), 2,3-di-*O*-methyl-galactose (R_t_: 17.61; *m/z* 118, 162, 261) and glucose (R_t_: 19.88; *m/z*, 145, 217, 289) indicative of Ser. 10–11. Isolates 3 and 8 had 2,3-di-*O*-methyl-fucose (R_t_: 12.88; *m/z* 118, 162, 203) indicative of Ser. 9. Isolates 4 and 6 were confirmed as Ser. 1, which is defined by containing the fragment ions of 6-deoxy-talose and rhamnose (R_t_: 15.01 and 15.12, respectively; and both with *m/z* 129, 171, 231), with their 3-*O*-methylated forms (R_t_: 14.06 and 14.4, respectively; and both with *m/z* 130, 143, 190). Isolates 5, 13 and 15 had the fragment ions of 2-*O*-methyl-fucose (R_t_: 14.04; *m/z* 118/275) and 4-*O*-methyl-rhamnose (R_t_: 14.41; *m/z* 131, 202, 262) indicative of Ser. 4. Isolates 7, 9, 10, 11, 12 and 14 had 3-*O*-methyl-glucose (R_t_: 18.99; *m/z* 130, 190, 261), which is characteristic for Ser. 8. The reference isolate 104 had the fragment ions of rhamnose, 6-deoxy-talose and their 3-O-methylated forms that defines Ser. 1 as above described. These analyses enabled us to conclude that isolate 2 was not a *M. avium* sp. strain, and thus it was removed from the study.

**Figure 5:**
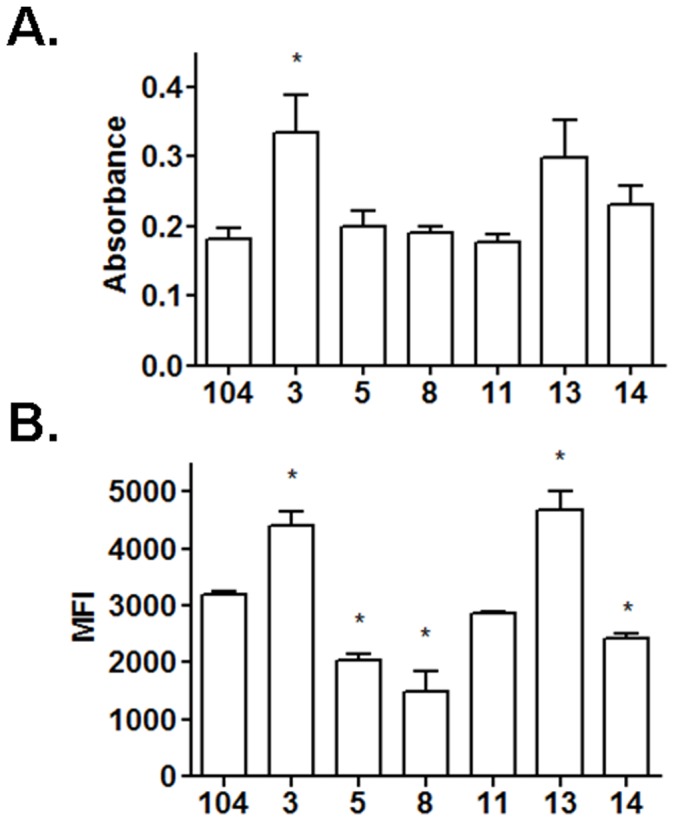
Detection of ManLAM on the bacterial surface. A. *M. avium* spp. isolates (2×10^6^/well) in a 96 well plate (triplicate wells per test group) were blocked with BSA and incubated for 2 h with CS-35 mAb followed by a secondary HRP-Ab. The graph shows cumulative data of N = 3 for selected samples (mean±SEM; *P<0.05). See table 4 for the complete set. **B.** Live bacilli single cell suspensions (1×10^6^) were blocked with 2% BSA in PBS, washed with PBS followed by staining with anti-LAM CS-35 in 2% BSA in PBS for 20 min at 4C. After further washing, samples were analyzed by flow cytometry. Mean fluorescence intensity (MFI) was measured for each isolate and the average values of 2 samples were obtained. Shown is cumulative data of N = 2 (mean±SEM). *P<0.05.

The combination of TLC and GC/MS results enabled the identification of the serovar for each isolate studied as depicted in Table 3. The results showed that Ser. 8 was predominant among the isolates studied; it was found in 6 of the 15 isolates (40%); Ser. 1 and 4 represented 5 of the total isolates (33%) and Ser. 9 was found in only 2 isolates (13%).

### Determination of ManLAM and LM on the Cell Wall of *M. avium* Spp. Isolates

To assess the total amount of lipoglycans in the cell wall of isolates, bacteria were lysed and equal amounts of protein (25 µg) analyzed by 15% SDS-PAGE followed by PAS staining and Western blotting using anti-LAM CS-35 mAb as we have previously described [29]. The results showed that all isolates studied contain ManLAM and LM (Fig. 4A). In addition, all ManLAMs were immune reactive by CS-35 mAb (especially strong in isolates 5, 6, 8, 9, 12, 13, 14 and reference 104) (Fig. 4B), although the presence of the CS-35 immune-reactive motif in the ManLAMs differed among the isolates.

We next determined by whole cell ELISA and flow cytometry whether ManLAM is exposed on the surface of the isolates and therefore potentially more readily recognized by macrophage surface receptors. The results indicated that ManLAM on MAC isolates 3, 4, 7, 9, 12, 13 and 15 were detected by CS-35, although reaching significance for only a few strains (Table 4 and Fig. 5) suggesting greater surface exposure of ManLAM. Isolates 5, 8 and 14 had no significant change in their ManLAM exposure from isolate 104 by ELISA and had a small but significant reduction by flow cytometry. The level of detection of total ManLAM in the cell wall of some of the isolates (Fig. 4) did not appear to correlate with the level of detection on the bacterial surface in all cases [*e.g.* 5, 6, 8 and 15 (Table 4 and Fig. 5)].

**Figure 6:**
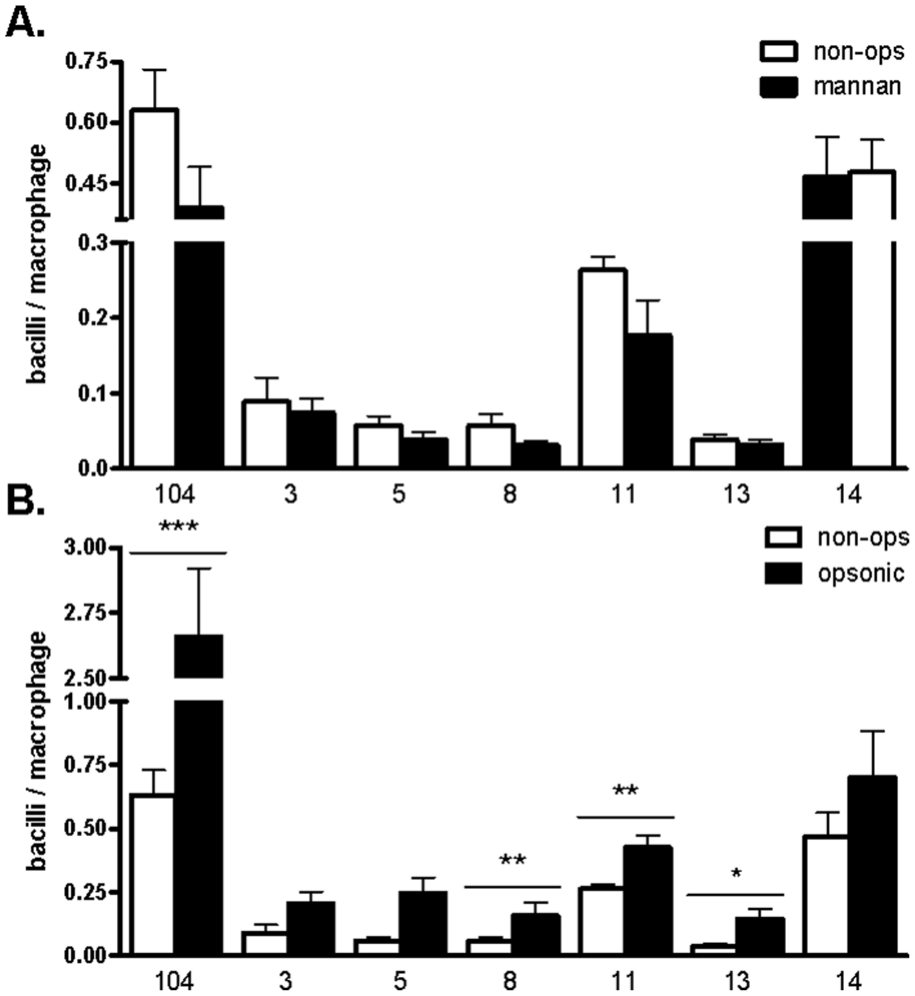
Association of *M. avium* spp. isolates with human macrophages. *A. Non-opsonic condition.* Macrophage monolayers (2.0×10^5^ cells/well) on glass coverslips in triplicate were incubated in the absence of serum with isolates (MOI 25∶1) for 2 h at 37°C, 5% CO_2_. MR-specific association was measured in the presence (close bars) of mannan (2.5 mg/ml). *B. Opsonic condition.* Macrophage monolayers were incubated with isolates as described above and association assessed in the absence (open bars) or presence (close bars) of 10% fresh autologous non-immune serum. Bacteria associated with macrophages were stained with rhodamine-auramine and association assessed by phase- contrast and fluorescence microscopy. >300 cells/coverslip were counted. Shown are cumulative data from N = 3 (mean±SEM) *P<0.05, *P<0.01, ***P<0.0001.

### Association of *M. avium* Spp. Isolates with Human Macrophages

To study the association of isolates with human macrophages, MDM monolayers were incubated with bacteria at an MOI 25∶1 for 2 h at 37°C in 5% CO_2_. Monolayers were washed, fixed and bacteria stained with auramine-rhodamine. Total isolate association (attached and ingested) was assessed by phase contrast and fluorescence microscopy. We selected a total of 6 representative isolates from our isolate pool (isolates 3, 5, 8, 11, 13 and 14) based on their content of surface-exposed ManLAM and total cell wall GPLs to perform this study. Isolate 3 was representative of an isolate with a high surface-exposed ManLAM (as determined by ELISA and flow cytometry) and lower cell wall GPL content (<60%); isolate 5 was representative of an isolate with low surface-exposed ManLAM and higher cell wall GPL content; isolate 8 was representative of an isolate with a low surface-exposed ManLAM and lower cell wall GPL content; isolate 11 was selected as it represented an isolate with similar ManLAM exposure and GPL content as the reference isolate MAC 104, isolate 13 was representative of an isolate with a high surface-exposed ManLAM and very low cell wall GPL content (<30%); and isolate 14 was representative of an isolate with a lesser surface-exposed ManLAM and lower cell wall GPL content. None of the clinical and environmental isolates analyzed had clearly defined high surface-expose ManLAM and high cell wall GPL content. The MAC 104 isolate was used as the reference isolate.

**Figure 7:**
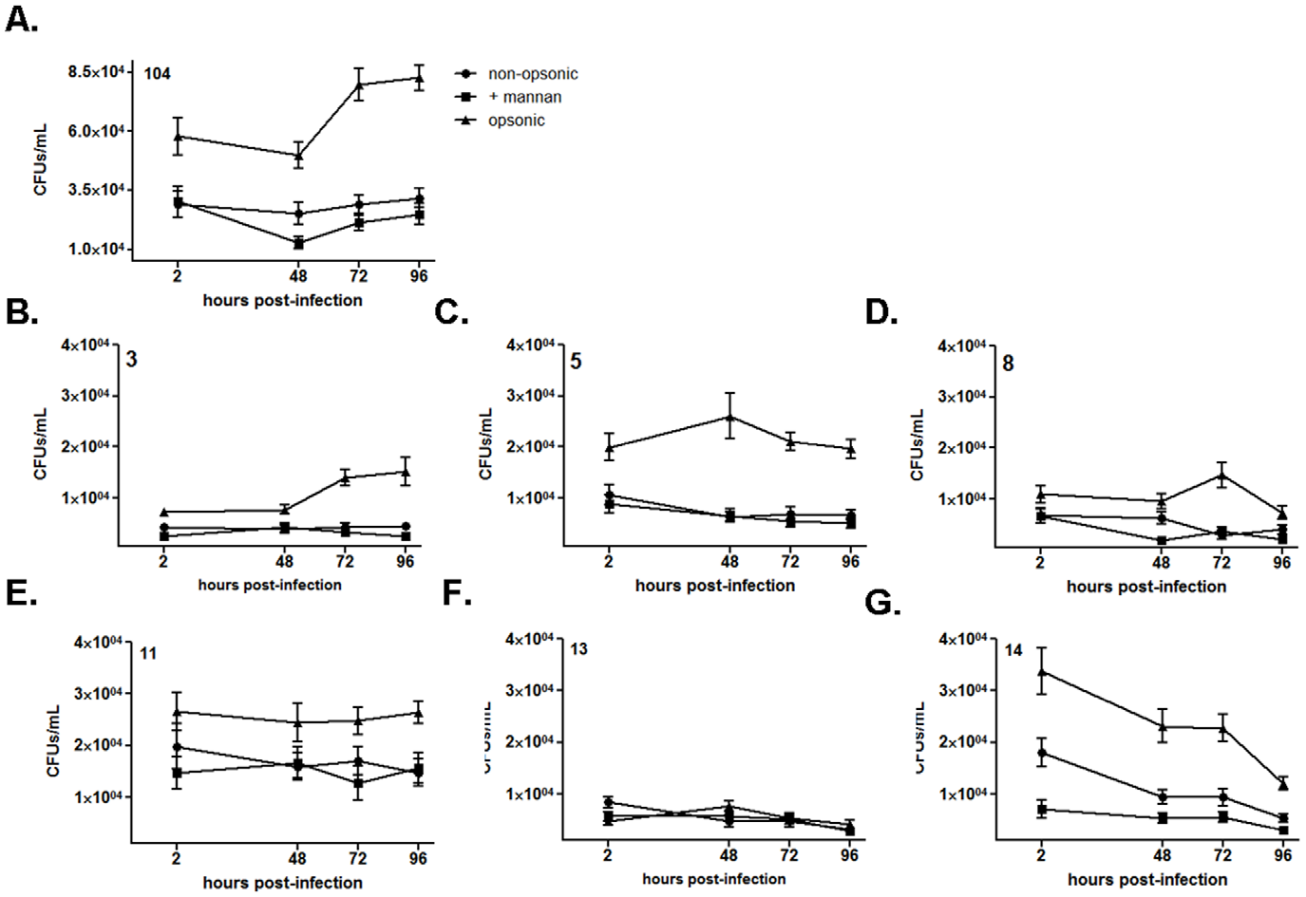
Growth of *M. avium* spp. isolates in human macrophages. Macrophage monolayers (2.0×10^5^ cells/well) plated in triplicate in cell culture plate wells were incubated with isolates (MOI 5∶1) for 2 h at 37°C, 5% CO_2_. Survival curves (CFUs) for MAC reference isolate 104 Ser. 1 (**A**), isolate 3 Ser. 9 (**B**), isolate 5 Ser. 4 (**C**), isolate 8 Ser. 9 (**D**), isolate 11 Ser. 8 (**E**), isolate 13 Ser. 4 (**F**) and isolate 14 Ser. 8 (**G**) are shown. Infection conditions used were: non-opsonic (round symbol); non-opsonic in the presence of mannan (2.5 mg/ml) (square symbol); and opsonic condition in the presence of 10% fresh autologous non-immune serum (triangle symbol). Note that the vertical axis in panel A differs from the other panels. Shown are cumulative data from N = 3 (mean ± SEM).

The results showed that MAC 104, isolate 11 and isolate 14 associated more with macrophages in all conditions studied (Fig. 6). The overall level of association was low, particularly in the absence of serum opsonins. The relative contribution of the macrophage MR was evaluated by adding the carbohydrate competitor mannan to block the interaction between the isolates and the MR in the absence of serum opsonins. Association of all isolates, except isolate 14, appeared to be MR-dependent, albeit to only a small extent (between 15–40%, Fig. 6A). To further assess the contribution of serum opsonins, we compared the level of association in the presence (opsonic conditions) or absence (non-opsonic conditions) of fresh autologous serum. Overall, as we previously described for *M.tb* and *M. leprae* [31], [34], in the presence of serum there was a 2–3 fold increase in bacterial association with human macrophages when compared to the no serum condition (Fig. 6B).

### Growth of *M. avium* Spp. Isolates in Human Macrophages

To assess whether different degrees of surface exposure of ManLAM and the content of GPLs affect the intracellular growth of bacteria in macrophages, we performed CFU assays. MDM monolayers were incubated with isolates in the presence or absence of mannan, and in the presence or absence of autologous serum, as described above for association studies. CFUs were enumerated at time 0 (2 h infection), 48 h, 72 h and 96 h post-infection. As above, we focused our attention on isolates 3, 5, 8, 11, 13 and 14, using the MAC isolate 104 as the reference isolate.

Results showed that the intracellular survival at 2 h post infection tended to correlate with the association values at 2 h. When the isolates were opsonized, the level of CFUs was increased at all time points relative to the non-opsonic condition. MAC 104 and isolate 3 showed growth over time following serum opsonization. The other isolates showed no growth or a reduction in growth over time (Fig. 7). Pre-incubation of macrophages with mannan had a small to moderate effect on CFUs indicative of a variable role for the MR in intracellular growth among the isolates. There was no apparent correlation with ManLAM exposure or GPL content in the CFU studies.

## Discussion

In this study we add new information to the growing body of evidence that GPLs and ManLAM can modulate cellular interactions. We demonstrate that clinical and environmental *M. avium* spp. isolates differ in their association and survival within human macrophages. However the differences seen do not clearly correlate with their cell envelope ManLAM surface exposure and ssGPL content.

The relation between MAC colony morphology and MAC virulence still remains unclear. It has been documented for decades that the MAC smooth, flat, transparent colony morphology observed on nutrient agar correlates with bacterial virulence and drug resistance, while smooth, domed, opalescent colonies correlate with an avirulent phenotype and drug susceptibility. Rough colonies on nutrient agar have been related to an attenuated strain phenotype [25], [26], [35]–[38], although some studies have shown that rough colonies can be as virulent as smooth colonies [25] or even more virulent [39], [40]. From the set of 15 *M. avium* spp. isolates studied only a few isolates produced a smooth transparent colony phenotype. Interestingly, the ratio of smooth *vs.* domed opaque colonies produced in all isolates studied was very low when compared to the reference 104 strain. Since isolates 3, 8 and 14 did not produce transparent colonies, and isolates 5, 11, 13 and MAC 104 produced transparent colonies, an attempt was made to establish some correlation between colony morphotype and virulence based on intracellular survival assays *in vitro* (for isolates 3, 5, 8, 11, 13 and 14); however, we found that there was no an obvious relationship. Surprisingly, rough or pinpoint colony morphotypes were not observed in any isolate studied as previously described for other MAC isolates [26].

Both TLC and GC/MS biochemical techniques, widely used in the field, were necessary to definitively establish the serovar of the set of *M. avium* spp. isolates studied. Tentative identifications using TLC were performed; however, this technique has a weakness related to its inability to distinguish among ssGPLs that have a similar mobility on TLC plates. Thus, the results combining TLC (as the initial screen) and GC/MS allowed us to identify the serovar for all *M. avium* spp. isolates studied. Among more than 31 serovars of MAC reported so far [16], Ser. 1, 4, 8 and 9 have been the most frequently isolated in AIDS patients around the world [1], [41], [42]. Specifically in the US, Ser. 4 and 8 are the predominant MAC serovars infecting humans, especially HIV-infected patients [43]. In this study using clinical and environmental *M. avium* spp. isolates provided by US EPA, Ser. 8 was the most predominant serovar found, followed by Ser. 4, Ser. 1 and Ser. 9.

We assessed the overall content and surface exposure of ManLAM, a mycobacterial lipoglycan that plays an important role in bacterial recognition and intracellular survival within human macrophages [22]. Our results (Fig. 4) indicate that *M. avium* spp. isolates 5, 6, 8, 9, 12, 13, 14 and reference 104 contain more ManLAM in their cell wall as detected by Western blot using anti-LAM CS-35 (MAC 104 is most often used as the reference strain because it has been sequenced [44], [45] and can be genetically manipulated [46]). We further assessed surface-exposed ManLAM using a whole cell bacterial ELISA and flow cytometry [29] since we predicted that these isolates would be better able to engage the MR pathway during the phagocytic process leading to increased intracellular survival. The amount of ManLAM (detected by Western blot) displayed by isolates 5, 6, 8 and 15 did not correlate with the exposure of this molecule on the bacterial surface as determined by ELISA and flow cytometry (Fig. 5). It is well known that GPLs represent the most abundant cell wall component of MAC. For isolates 5, 6 and 8 that have higher ManLAM content as determined by Western blot but low surface exposure (ELISA), it is possible that the GPLs are masking ManLAM to some extent leaving the latter less accessible on the surface of the bacteria to freely interact with the MR. Similarly we have found that some clinical isolates of *M.tb* have their ManLAM buried in the cell wall by other outer surface constituents [29].

We elected to focus our attention on isolates 3, 5, 8, 11, 13, 14 and reference 104 for macrophage association and survival studies based on their ManLAM exposure and GPL content. Despite having ManLAM readily exposed on their surface, isolates 3 and 14 did not show the expected level of engagement with the MR on macrophages as indicated by their limited to no inhibition of association in the presence of mannan (a competitor for the MR). Reference isolate 104 serovar 1 and clinical isolates 11 and 14 serovar 8 showed an increased association with human macrophages independent of the infection condition studied (opsonic *vs.* non-opsonic). Together with the survival studies our data support the notion that the amounts and spatial distribution of cell wall ManLAM and GPLs, as well as GPL serovar specificity do not alone dictate the early interactions of *M. avium* with human macrophages.

It is difficult to predict the true virulence properties of a MAC isolates based on the outcome observed *in vitro* as these results may not mirror the expected findings in animal models [37], [47]. However, animal models are not always predictive of outcomes in humans [48]. There is a need for new screening tools that can identify specific virulence properties of MAC isolates that predict a level of risk to exposed individuals [49]. Screening approaches that include biochemical characterization of MAC isolate phenotypes, their interaction with human macrophages and virulence *in vivo* may provide us with a better knowledge of the capacity of a specific environmental isolate to be pathogenic in predisposed human populations.
